# How and when does monkeypox (mpox) transmit: Implications for prevention and treatments

**DOI:** 10.3389/fphar.2022.1109928

**Published:** 2023-01-05

**Authors:** Shu Yuan, Si-Cong Jiang, Zhong-Wei Zhang, Yu-Fan Fu, Xin-Yue Yang, Zi-Lin Li, Jing Hu

**Affiliations:** ^1^ College of Resources, Sichuan Agricultural University, Chengdu, China; ^2^ Haisco Pharmaceutical Group Comp., Ltd., Chengdu, China; ^3^ Department of Cardiovascular Surgery, Xijing Hospital, Medical University of the Air Force, Xi’an, China; ^4^ School of Medicine, Northwest University, Xi’an, China

**Keywords:** monkeypox virus, mild-symptomatic patients, asymptomatic patients, seminal transmission, viral load

## Introduction

In this year, more than 70,000 mpox cases in non-endemic countries around the world have been reported, most of which were in American and Europe. Monkeypox virus (MPXV) is mainly transmitted by direct contact, including close contacts with skin lesions, respiratory secretions, or contaminated items of infected patients or animals (Bunge et al., 2022; Perez Duque et al., 2022). MPXV infection outbreak usually has a central point, and the original patient should have travelled to epidemic areas or have a clear history of exposure to infectious sources (such as some animals; Bunge et al., 2022). However, the current outbreak occurs in several non-endemic countries simultaneously, and the most reported cases have neither contacted with wild animals directly nor been to the endemic countries in Africa (Perez Duque et al., 2022; Saied et al., 2022). Moreover, after the Corona Virus Disease 2019 (COVID-19), people’s social distance increases, and the probability of contact transmission was decreased. It is difficult to explain the current mpox epidemic with the common transmission pathways (Saied et al., 2022).

In our previous study (Yuan et al., 2022), through cluster analysis of MPXV based on relative synonymous codon usage (RSCU) bias, we concluded that the current mpox outbreak in American and Europe may have at least three origins: Sudan 2005—Nigeria 2017 cluster, Sierra Leone 2004 cluster, and Libya 1970 cluster. The geographical distribution of viral clusters was in cross, implying that they were multi-originated and the transmission paths might be very complex (Yuan et al., 2022).

Before this year, mpox was not listed as a sexually transmitted disease (STD). For the current outbreak, most mpox patients were gay, bisexual, and other men who have sex with men (MSM) with sex tourisms (Thornhill et al., 2022a; Thornhill et al., 2022b; Patel et al., 2022). However, for a contagious STD, the median incubation period was only about 7–9 days (Thornhill et al., 2022a; Guzzetta et al., 2022; Miura et al., 2022; Ward et al., 2022), which may be too short to cause a large-scale transmission (the incubation period of HIV was about 10 years; Román-Montoya et al., 2013). The unexpected and sudden appearance of MPXV concurrently in several non-endemic areas indicates that there may be some unnoticed transmission in some unknown duration of time followed by recent amplifier events (Alakunle and Okeke, 2022).

## High ratio of mpox-HIV co-infection

A large number of mpox patients had concomitant HIV infection with a ratio of 42.2% (78/185; Català et al., 2022), 35.9% (70/195; Patel et al., 2022) or 41.3% (218/528; Thornhill et al., 2022a) respectively. Although most mpox patients were MSM (Thornhill et al., 2022a; Thornhill et al., 2022b; Patel et al., 2022), the ratios of mpox-HIV co-infection were much higher than the usual percentage of HIV diagnoses in MSM (<2%; Rao et al., 2016).

We noticed that only 8% of the patients showed detectable HIV viral loads (Català et al., 2022). Other two reports also demonstrated that 78.6% (55/70; Patel et al., 2022) or 97.4% (185/190; Thornhill et al., 2022a) patients with mpox-HIV co-infection had low HIV viral loads (<200 copies/mL). All these data suggested that HIV-positive population in mpox patients showed very good HIV control. Therefore, they were individuals living with HIV infection (but not HIV clinics with symptoms) and more likely to have high-risk sexual behaviors.

Secondly, in HIV patients, some clinical characteristics of mpox might be different from non-those in non-HIV patients (Amorosa and Isaacs, 2003; Saied et al., 2022). Although in general, well-controlled HIV was not associated with severity of the symptoms, HIV-positive patients were more likely to have fevers (60% of HIV patients vs. 50% of non-HIV patients; Català et al., 2022). And the HIV-positive patients tended to show larger numbers of lesions or affected areas (Català et al., 2022). In non-HIV infected cases, the patients usually present with generalized skin rash. For the HIV infected cases, there might be the more skin lesion at genital or perinatal areas (Hammerschlag et al., 2022; Mungmunpuntipantip and Wiwanitkit, 2022). In a retrospective review of hospital records of 40 human mpox cases from Nigeria, the HIV type 1-coinfected cases showed more prolonged illness, larger lesions, and higher rates of both secondary bacterial skin infections and genital ulcers (Ogoina et al., 2020). Severe symptoms after poxvirus infections may develop in immuno-compromised individuals (Amorosa and Isaacs, 2003). So HIV-positive patients were more likely to go to the hospital, although they might seek dermatovenerologic diagnosis prior to visiting other specialists (Hammerschlag et al., 2022). A study reported that, of 20 participants admitted to hospital for clinical reasons, 15 (75.0%) had HIV co-infection (Patel et al., 2022).

## The role of mild-symptomatic patients in unnoticed mpox transmission

Thornhill et al. (2022a) demonstrated that the median incubation period of mpox was about 7 (3–20) days. However, longer mean incubation periods have also been reported, which were estimated to be 7.6–7.8 days (95% credible interval 6.5 to 9.9; Ward et al., 2022), 8.5 days (95% credible interval 4.2 to 17.3; Miura et al., 2022) or 9.1 days (95% credible interval 6.5 to 10.9; Guzzetta et al., 2022). The difference in incubation period may be attributed into different definition to the symptom onset. Usually, the definition of symptom onset describes the date that an individual first noticed their symptoms. However, the initial appearance after mpox virus (MPXV) infection may be just atypical (mild) genital and peri-anal rashes without severe pain (Thornhill et al., 2022a; Thornhill et al., 2022b; Patel et al., 2022; Tarín-Vicente et al., 2022). Thus, the true date of symptom onset may be earlier but not detected.

Ward et al. (2022) found that short serial intervals were more common than short incubation periods, therefore suggesting a considerable pre-symptomatic transmission. Nevertheless, the genital or rectal lesion swabs obtained from mpox patients only became positive for MPXV DNA until after 3–5 days post symptom onset (Table 1). In other words, most pre-symptomatic patients may be not infectious. The term “pre-symptomatic transmission” may be inaccurate and should be interpreted as “mild-symptomatic transmission.”

**TABLE 1:** Timeline of PCR results from mpox cases in 2022.

Time of the first positive PCR result	Sampling site	PCR Ct value	Sample size (*n*)	References
5 (2–20) days after symptom onset (dso)	Skin or anogenital lesion (97%)	≤40	528	Thornhill et al. (2022a)
	Nose or throat swab (26%)			
	Blood (7%)			
	Urine (3%)			
	Semen (5%)			
5 dso	Serum	29.7	4	Antinori et al. (2022)
5 dso	Plasma	30.2		
3–5 dso	Genital or rectal lesions	14.7–17.5		
3–5 dso	Nasopharyngeal swab	27.6–30.4		
3–5 dso	Skin lesions	17.6–30.4		
5–9 dso	Seminal fluid	27.7–43.2		
5 dso	Scab	13.1–20.0		
3–6 dso	Faeces	22.6–26.1		
3 dso	Saliva	27.1		
Asymptomatic stage (-7—-9 dso)	Anal swabs	20.7–38.2	2	Ferré et al. (2022)
Presymptomatic patients	Anorectal swab	17.16–26.69	3	De Baetselier et al., 2022; Van Dijck et al., 2022
7.0 (5.0–10.0) dso	Skin swab (99%)	23	180	Tarín-Vicente et al. (2022)
	Throat swab (70%)	32	117	
	Anal swab (78%)	27	55	
4–16 dso	Saliva	20.3–37.9	22	Peiró-Mestres et al. (2022)
4–14 dso	Rectal swab	17.6–38.4	23	
4–14 dso	Nasopharyngeal swab	25.4–40.0	23	
1–14 dso	Semen	22.7–40.0	16	
1–16 dso	Urine	24.4–40.0	23	
4–16 dso	Faeces	19.9–31.4	22	
3–6 dso	Skin lesions	17–27	4	Hornuss et al. (2022)
3–9 dso	Nasopharyngeal swab	28–35		
4–9 dso	Anal mucosa	23–31		
3–11 dso	Blood	30–39		
4–9 dso	Urine	34–38		
14	Seminal fluid	33-38		

The mild-symptomatic patients may play a key role in the early unnoticed transmission, because that the individuals may still be engaged in high-risk sexual behaviors in the first few days post symptom onset. The genital and peri-anal rashes may be rubbed raw during the sexual intercourse and the virus would be released. Then MPXV may get into the blood stream directly, if anal bleeding occurs. A case study reported a MPXV transmission to a healthcare worker through a needlestick injury, confirming a possibility of direct blood transmission (Carvalho et al., 2022).

## Possible seminal transmission of MPXV

MSM are prone to have condomless sexual intercourse and leave the seminal fluid inside the body. Before this year, mpox was not known as a sexually transmitted disease. MSM usually adopt HIV pre-exposure prophylaxis (PrEP; Hodges-Mameletzis et al., 2019; Atim et al., 2020; Thornhill et al., 2022a). However, use of PrEP may be a risk factor for MPXV infection, because that MSM with PrEP do not often use condoms (Torster et al., 2022). WHO recommended PrEP since 2015 (Hodges-Mameletzis et al., 2019; Atim et al., 2020). Thus, the current correlation between sexual behaviors and MPXV infections found in this year might be explained.

The available literatures showed increasing concerns about possible seminal transmission of MPXV (Hornuss et al., 2022; Lapa et al., 2022; Noe et al., 2022; Peiró-Mestres et al., 2022; Raccagni et al., 2022; Reda et al., 2022; Reda et al., 2023). Detection of viruses in the testes is commonly secondary to viraemia because the blood–testis barrier may be liable to viruses, especially when systemic or local inflammation occurs. Viral persistence through the tract is also likely, no matter of its capability to replicate, because the testis can be an immunological-favored site for viruses (Li et al., 2012; Annandale et al., 2014; Mead et al., 2018). Interestingly, culturing MPXV was successful in two out of four patients included in two studies (Lapa et al., 2022; Noe et al., 2022), suggesting a replication competence of MPXV detected in seminal specimens.

A clinical study reported positive MPXV results in the seminal fluid obtained from mpox patients at the time closest (5–7 days) to symptoms onset with a Ct range from 27 to 30 (Antinori et al., 2022); when the symptoms may be mild. Though in a low viral load, seminal MPXV may be still contagious. Alternatively, seminal MPXV may get into the blood stream directly, if anal bleeding occurs.

## Asymptomatic patients might transmit the virus through seminal fluids

Asymptomatic mpox infections may be observed in both smallpox vaccinated and unvaccinated individuals (Karem et al., 2007; Guagliardo et al., 2020). Ferré et al. (2022) detected MPXV in anorectal swabs from asymptomatic MSM. Among 200 participants who were subjected to MPXV PCR tests, they reported 13 MPXV-positive participants who were initially asymptomatic (two of them showed mild symptoms 7–9 days later). However, asymptomatic patients do not develop rashes or skin lesions, where the viral loads are the highest (about 10,000 times higher than in serum; Table 1). Therefore they are believed to be of little or no epidemiologic importance. Nevertheless, Ferré et al. (2022) also found a high viral load in a patient during the asymptomatic stage with a very low Ct value of 20.7. And serology confirmed that MPXV isolated from two presymptomatic cases can be cultured (De Baetselier et al., 2022; Van Dijck et al., 2022). Whether a high viral load in seminal fluid obtained from some asymptomatic patients could be detected needs further investigations. There might be a possibility that asymptomatic patients transmit the virus through seminal fluids.

## Condom, vaccines and drugs

The condom could prevent direct contact with anogenital lesions, where the viral loads are the highest (Table 1). Although the actual protection rate of condoms against mpox infection is unclear, compared with the vaccines and drugs, use of condoms may be the most effective and convenient way to control the current epidemic.

Given that in most cases, the viral load peaks after 3–5 days post symptom onset (Table 1), vaccination and/or drug treatments before this time-point may show good therapeutic effects. All highly-susceptible populations should be subjected to viral tests and priority treatments, no matter in symptomatic or asymptomatic, especially for those are too young to receive childhood smallpox vaccination, whose viral loads may be higher than unvaccinated people. However a large part of them had concomitant HIV infection (Thornhill et al., 2022a; Català et al., 2022; Patel et al., 2022). Previous studies suggested that HIV-positive individuals with CD4 cell counts of <300 cells/mm^3^ may develop severe complications after vaccinia virus vaccination (Amorosa and Isaacs, 2003). Thus, for those with low CD4 cell counts, the decision whether or not to vaccinate must be made within the context and circumstances of the mpox outbreak. Alternatively, the immuno-compromised people or the patients with atopic dermatitis should receive a third-generation non-replicating vaccine that was made based on modified vaccinia Ankara (MVA) (Saied et al., 2022). It is interesting to note that some MVA vaccine may be considered for post-exposure prophylaxis, ideally within 4 days of high-grade exposure (Vaughan et al., 2020).

The mainstay of clinical treatments for MPXV infections are supportive and/or symptomatic managements (Reynolds et al., 2017). Although there are a few antiviral drugs have been prescribed for mpox patients, such as Cidofovir, Brincidofovir, and Tecovirimat (Adler et al., 2022; Thornhill et al., 2022a; Rizk et al., 2022; Saied et al., 2022), no prophylactic drug has been approved. Whether some drugs could be considered in mpox pre-exposure prophylaxis needs further investigations. Besides above vaccines and drugs, Saied et al. (2022) further suggested that vaccinia immune globulin intravenous (VIGIV) or vaccine immune globulin (VIG) may be used for mpox treatments, and especially helpful to the immuno-compromised people, pregnant women, or the patients with complicated lesions.
